# Restoration of primary cilia in obese adipose-derived mesenchymal stem cells by inhibiting Aurora A or extracellular signal-regulated kinase

**DOI:** 10.1186/s13287-019-1373-z

**Published:** 2019-08-14

**Authors:** Andreas Ritter, Nina-Naomi Kreis, Susanne Roth, Alexandra Friemel, Lukas Jennewein, Christine Eichbaum, Christine Solbach, Frank Louwen, Juping Yuan

**Affiliations:** Department of Gynecology and Obstetrics, School of Medicine, J. W. Goethe-University, Theodor-Stern-Kai 7, D-60590 Frankfurt, Germany

**Keywords:** Adipose-derived mesenchymal stem cells, Primary cilium, Obesity, Aurora A, Extracellular signal-regulated kinase 1/2, Glycogen synthase kinase 3 beta

## Abstract

**Background:**

Obesity impairs a variety of cell types including adipose-derived mesenchymal stem cells (ASCs). ASCs are indispensable for tissue homeostasis/repair, immunomodulation, and cell renewal. It has been demonstrated that obese ASCs are defective in differentiation, motility, immunomodulation, and replication. We have recently reported that some of these defects are linked to impaired primary cilia, which are unable to properly convey and coordinate a variety of signaling pathways. We hypothesized that the rescue of the primary cilium in obese ASCs would restore their functional properties.

**Methods:**

Obese ASCs derived from subcutaneous and visceral adipose tissues were treated with a specific inhibitor against Aurora A or with an inhibitor against extracellular signal-regulated kinase 1/2 (Erk1/2). Multiple molecular and cellular assays were performed to analyze the altered functionalities and their involved pathways.

**Results:**

The treatment with low doses of these inhibitors extended the length of the primary cilium, restored the invasion and migration potential, and improved the differentiation capacity of obese ASCs. Associated with enhanced differentiation ability, the cells displayed an increased expression of self-renewal/stemness-related genes like *SOX2*, *OCT4*, and *NANOG*, mediated by reduced active glycogen synthase kinase 3 β (GSK3β).

**Conclusion:**

This work describes a novel phenomenon whereby the primary cilium of obese ASCs is rescuable by the low-dose inhibition of Aurora A or Erk1/2, restoring functional ASCs with increased stemness. These cells might be able to improve tissue homeostasis in obese patients and thereby ameliorate obesity-associated diseases. Additionally, these functionally restored obese ASCs could be useful for novel autologous mesenchymal stem cell-based therapies.

**Electronic supplementary material:**

The online version of this article (10.1186/s13287-019-1373-z) contains supplementary material, which is available to authorized users.

## Background

Obesity is one of the most serious and fastest-growing health problem in the world [1]. It significantly increases the risk for the development of multiple disorders including cardiovascular diseases and various cancer entities [2–5]. During the development of obesity, a dramatic remodeling of the adipose tissue is taking place characterized by infiltration of numerous inflammatory immune cells, highly enhanced secretion of pro-inflammatory cytokines/chemokines, and reduced angiogenesis [6, 7]. Despite intense research over the last decade, the molecular mechanisms underlying the pathogenesis of obesity and its related diseases are not completely understood.

Adipose-derived mesenchymal stem cells (ASCs) are a key component of the adipose tissue, responsible for adipogenesis, immunomodulation, tissue repair, and angiogenesis [8, 9]. As reported recently, obese ASCs have a decreased differentiation capacity, an altered adipokine/chemokine secretion, and reduced migration [3, 9–11]. Defective ASCs might contribute to the development of obesity and its related diseases by interfering with adipose tissue remodeling, fueling the pro-inflammatory milieu, and deteriorating hypoxia [3, 12].

We have recently shown that obese ASCs have defective primary cilia [11]. Primary cilia are microtubule-based organelles protruding from the surface of almost all vertebrate cells. They conduct a multitude of different signals from the extracellular environment via diverse receptors regulating the cell cycle, cell growth, development, and cellular homeostasis [13, 14], turning them into central platforms for cell-cell interaction and signaling [15]. Defects or a complete loss of cilia lead to severe human developmental disorders known as ciliopathies [16]. Moreover, the primary cilium specifically transduces Hedgehog (Hh) signaling [15] and is involved in maintaining self-renewal and differentiation of ASCs [9, 17, 18].

By investigating the causes of impaired cilia, we found that inflammatory cytokines such as interleukin 6 (IL6) or tumor necrosis factor α (TNFα) are capable of triggering this impairment in ASCs [9, 11]. Our findings implicate an association between the development of obesity and the loss of functional ASCs. We assume that the stabilization of the primary cilium could restore the functionalities of obese ASCs. Indeed, in the present work we show that the treatment with low dose of the inhibitor MLN8054 (MLN) against the Aurora A kinase or PD98059 (PD) against extracellular signal-regulated kinase 1/2 (Erk1/2) rescues the length and functionality of primary cilia of obese ASCs, accompanied by increased levels of the genes related to self-renewal/stemness. These findings might be of clinical importance in terms of autologous mesenchymal stem cell-based therapies.

## Methods

### Human ASC isolation, surface marker measurement, and reagents

This work was approved by the Ethics Committee of the Johann Wolfgang Goethe University Hospital Frankfurt, and informed written consent was obtained from all participants. Visceral (omental) and subcutaneous (abdominal) adipose tissues were taken from women undergoing a cesarean section. Participant information is listed in Additional file 1: Table S1. ASCs were isolated as described [19, 20]. Cells were cultured and expanded for three passages. Cells were then stored at − 80 °C until use. Early passages (P3 to P6) of ASCs were used for all experiments. FACSCalibur™ (BD Biosciences, Heidelberg) was used for determining the surface markers of ASCs. Cells were harvested with 0.25% trypsin, fixed for 15 min with ice-cold 2% PFA at 4 °C. Cells were washed twice with flow cytometry buffer (FCB: PBS with 0.2% Tween-20 and 2% FCS) and stained with the following antibodies from eBioscience/BD-Pharmingen (Frankfurt): FITC-conjugated anti-human CD90 (#11-0909-42), PE-conjugated anti-human CD73 (#550257), PE-conjugated anti-human CD 105 (#323206), PE-conjugated anti-human CD146 (#561013), PerCP-Cy5.5-conjugated anti-human CD14 (#555397), FITC-conjugated anti-human CD34 (#343504), APC-conjugated anti-human CD106 (#551147), and APC-conjugated anti-human CD31 (#17-0319-41). Anti-mouse Ig, κ/negative control compensation particles (eBioscience/BD-Pharmingen, #552843), flow cytometry setup beads (eBioscience/BD-Pharmingen, #340486 and #340487), and non-stained ASCs were used as negative controls for FACS gating.

IL6 and TNFα were from PeproTech (Hamburg). Aurora A inhibitor MLN8054, Erk1/2 inhibitor PD98059, GSK3β inhibitor CHIR99021, and PI3K inhibitor Wortmannin were obtained from Sigma-Aldrich (Taufkirchen). Plk1 inhibitor BI 6727 was from Selleckchem (Munich).

### Indirect immunofluorescence staining, microscopy, and intensity measurement

Indirect immunofluorescence staining was performed as reported [21, 22]. Cells were seeded on Nunc™ Lab-Tek™ SlideFlask chambers from Thermo Fisher Scientific (Schwerte). Cells were fixed for 8–10 min with methanol at − 20 °C or with 4% paraformaldehyde containing 0.2% Triton X-100 for 15 min at room temperature as described [21, 22]. The following primary antibodies were used: rabbit polyclonal antibody against pericentrin (Abcam, Cambridge, #AB28144), mouse monoclonal antibody against acetylated α-tubulin (Sigma-Aldrich, #T6793), mouse monoclonal antibody against Smo (Santa Cruz Biotechnology, #sc-166,685), rabbit polyclonal antibody against Arl13b (Proteintech, Herford, #17711-1-1AP), mouse monoclonal antibody against Aurora A (Cell Signaling, Frankfurt am Main, #12100), rabbit monoclonal antibody against phospho-Aurora A (Thr288) (Cell Signaling, #3079), and rabbit polyclonal antibody against phospho-histone H3 (pHH3, Ser10, Merck Millipore, Darmstadt, #06-570). FITC-, Cy3-, and Cy5-conjugated secondary antibodies were obtained from Jackson ImmunoResearch. DNA was visualized by using DAPI (4′,6-diamidino-2-phenylindole-dihydrochloride, Roche, Mannheim). Slides were examined using an AxioObserver.Z1 microscope (Zeiss, Göttingen), and images were taken using an AxioCam MRm camera (Zeiss). The immunofluorescence-stained slides were further examined by confocal laser scanning microscopy (CLSM) using Z-stack images with a HCXPI APO CS 63.0 × 1.4 oil objective (Leica CTR 6500, Heidelberg) in sequential excitation of fluorophores. A series of Z-stack images were captured at 0.5-μm intervals. All images in each experiment were taken with the same laser intensity and exposure time. All experiments, unless otherwise indicated, were independently performed with ASCs isolated at least from three different donors. Representatives are generated by superimposing (overlay) individual images from confocal Z-sections.

Fluorescent intensity was measured using line-scan-based analysis via ImageJ (National Institutes of Health), as described [11, 23]. The average intensities over a three-pixel-wide line along the axoneme were measured and normalized against cilium length by using the ImageJ plugin Plot Roi Profile. The intensity was measured from the axonemal base to its tip in 10% intervals. The mean values of 30 cilia from three different donors were obtained for each group within the intervals and were plotted to GraphPad Prism 7 (GraphPad Software Inc.).

### SAG stimulation, cell cycle analysis, and ELISA

For activating the Hh pathway, cells were incubated with 200 nM SAG (Bioscience, Wiesbaden) in the absence of FCS for 24 h. Immunofluorescence line-scan-based analysis and quantitative RT-PCR analysis were then performed. The cell cycle distribution was analyzed using a FACSCalibur™ (BD Biosciences), as reported [24]. Briefly, cells were harvested, washed with PBS, fixed in chilled 70% ethanol at 4 °C for 30 min, treated with 1 mg/ml of RNase A (Sigma-Aldrich), and stained with 100 μg/ml of propidium iodide (PI) for 30 min at 37 °C. DNA content was determined. Seventy-two-hour supernatants were collected before and after differentiation for evaluating IL6 and TNFα (PeproTech, Hamburg) and adiponectin (Sigma-Aldrich) via ELISA as instructed by the manufacturers.

### ASC differentiation and Western blot analysis

ASC differentiation was performed as reported [19]. To induce adipogenic differentiation, ASCs were cultured with StemMACS AdipoDiff media (Miltenyi Biotec, Gladbach) up to 14 days. Cells were then fixed and stained for oil red O and adiponectin (Abcam, Cambridge, #AB22554) characteristic of adipocytes. For osteogenic differentiation, ASCs were incubated in StemMACS OsteoDiff media (Miltenyi Biotec) up to 14 days, fixed, and stained with 2% alizarin red S (pH 4.2) to visualize calcific deposition in cells of an osteogenic lineage. Western blot analysis was performed as reported [21, 24], using rabbit monoclonal antibodies against p44/42 Erk1/2 (#9102), rabbit polyclonal phospho-p44/42 Erk1/2 (Thr202/Tyr204) (#9101), rabbit monoclonal GSK3β (27C10) (#9315), rabbit polyclonal phospho-GSK3β (Ser 9) (#9331), mouse monoclonal STAT3 (124H6) (#9139), rabbit monoclonal pSTAT3 (Tyr705) (#9313), mouse monoclonal β-actin (A2228) (Sigma-Aldrich), and GAPDH (#MA5-15738) from ThermoFisher Scientific (Frankfurt).

### RNA extraction and real-time PCR

Total RNAs of ASCs were extracted with RNeasy Mini kit (QIAGEN, Hilden). Reverse transcription was performed using High-Capacity cDNA Reverse Transcription Kit (Applied Biosystems, Darmstadt), as instructed. All probes for gene analysis were obtained from Applied Biosystems: *ADIPOQ* (#Hs00605917_m1), *AURKA* (#Hs01582072_m1), *AURKB* (#Hs00945858_g1), *CCP110* (#Hs00206922_m1), *PLK1* (#Hs00153444_m1), *PLK4* (#Hs00179514_m1), *KIF2A* (#Hs00189636_m1), *KIF24* (#Hs00950248_m1), *HDAC6* (#Hs00195869_m1), *SMO* (#Hs01090242_m1), *GLI1* (#Hs00171790_m1), *NANOG* (#Hs04260366_g1), *PTCH1* (#Hs00181117_m1), *RUNX2* (#Hs01047973_m1), *KLF4* (#Hs00358836_m1), *c-MYC* (#Hs00153408_m1), *KLF6* (#Hs00810569_m1), *PPARγ* (#Hs01115513_m1), *LEPTIN* (#Hs00174877_m1), *OCT4* (#Hs04260367_gH), *SOX2* (#Hs01053049_s1), and *GAPDH* (#Hs02758991_g1). Real-time PCR was performed with a StepOnePlus Real-time PCR System (Applied Biosystems). The data were analyzed using StepOne Software v.2.3 (Applied Biosystems) as described previously [11].

### Cell motility, migration, and invasion

Cells were seeded into 24-well plates with a low confluency and were imaged for 12 h at 5-min time intervals. All time-lapse imaging was performed with an AxioObserver.Z1 microscope (Zeiss), imaged with an AxioCam MRc camera (Zeiss) equipped with an environmental chamber to maintain proper environmental conditions (37 °C, 5% CO_2_). The time-lapse movies were analyzed by using ImageJ 1.49i software (National Institutes of Health) with the manual tracking plugin, and Chemotaxis and Migration Tool (Ibidi GmBH, Munich). Tracks were derived from raw data points and were plotted in GraphPad Prism 7 (GraphPad Software Inc.). The accumulated distance was calculated by using the raw data points by the Chemotaxis and Migration Tool. Thirty random cells per experiment were analyzed, and the experiments were repeated independently three times. The patterns of motility were evaluated as described previously [11, 20, 25].

Cell migration assays were performed with culture-inserts from ibidi (Martinsried). Visceral or subcutaneous ASCs (6.5 × 10^4^) were seeded in each well of the culture inserts. Culture inserts were gently removed after at least 8 h. The cells were acquired and imaged at indicated time points with bright-field images. Four pictures of each insert were taken (three inserts for each experimental condition), and the experiments were performed in triplicates. The open area was measured using the AxioVision SE64 Re. 4.9 software (Zeiss).

For invasion assay, visceral or subcutaneous ASCs were seeded (7.5 × 10^4^) in 24-well transwell matrigel chambers according to the manufacturer’s instructions (Cell Biolabs Inc., San Diego) and as previously reported [26]. Cells were fixed with ethanol and stained with DAPI. Invaded cells were counted with a microscope. The experiments were independently performed three times.

### Statistical analysis

Student’s *t* test (two-tailed and paired or homoscedastic) was used to evaluate the significance of the difference between diverse groups for gene analysis, cell viability assay, cell cycle distribution, and ciliated cell population. The statistical evaluation of the single-cell tracking assay, line-scan analysis, and the measurement of the cilium length was performed by using an unpaired Mann-Whitney *U* test (two-tailed). The difference was considered statistically significant when *p* < 0.05.

## Results

### Low dose of Aurora A or Erk1/2 inhibitor restores the cilium length in obese ASCs

**Fig. 1:**
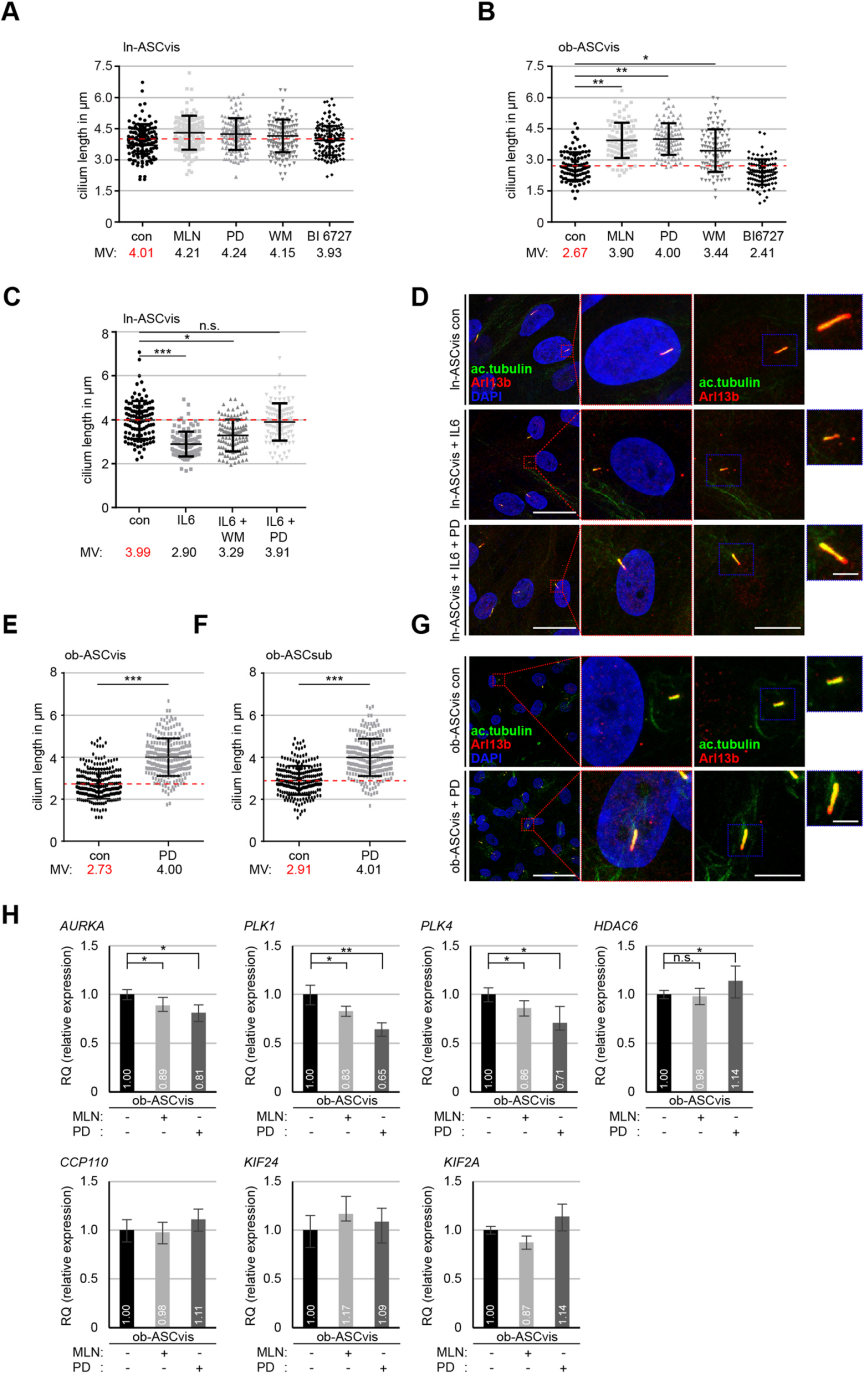
Treatment with MLN8054 or PD98059 rescues cilium length and reduces the expression level of deciliation genes like *Aurora A*, *PLK1*, and *PLK4* in obese ASCs. **a**, **b** The cilium length was measured in visceral ln-ASCs and ob-ASCs treated with MLN8054 (MLN, 15 nM), PD98059 (PD, 25 nM), Wortmannin (WM, 15 nM), and BI 6727 (15 nM). The results are based on three experiments using ASCs from three obese and three lean donors (*n* = 100 cilia for each group) and presented as scatter plots. Red dashed line indicates the cilium mean length of control cells. **c** Evaluation of the cilium length in visceral ln-ASCs treated with IL6 or IL6 in combination with WM or PD. The results are based on three experiments using ASCs from three lean donors (*n* = 100 cilia for each group). **d**, **g** Lean (D) or obese (G) visceral ASCs were stained as indicated. Representatives are shown. Scale bar, 30 μm. Magnified representatives scale bar, 4 μm. Inset scale bar, 3 μm. **e**, **f** Quantification of visceral and subcutaneous obese ASCs treated with PD inhibitor. The results are based on six experiments using ASCs from six obese donors (*n* = 230 cilia for each group). **h** The gene levels of deciliation molecules (*AURKA*, *PLK1*, *PLK4*, *HDAC6*, *CCP110*, *KIF24*, and *KIF2A*). The data are based on three experiments and presented as mean ± SEM. RQ, relative quantification of gene expression. Scatter plots were used to show the mean and the minimal to maximal range of the values in **a**–**f**. Unpaired Mann-Whitney *U* test for **a**, **b**, **c**, **e**, and **f**. Student’s *t* test for **h**. ∗*p* < 0.05, ∗∗*p* < 0.01, ∗∗∗*p* < 0.001

Ciliogenesis is tightly associated with the cell cycle [13] and the assembly as well as the disassembly of the primary cilium depend on a variety of kinases like Aurora A and Polo-like kinase 1 (Plk1) [16]. To test whether and to which extent kinase inhibitors are able to rescue the ciliary length of obese ASCs, ln-ASCs (BMI < 25, ln-ASCs, from healthy lean control donors) and ob-ASCs (BMI > 35, ob-ASCs, from obese donors) were treated with MLN8054 (MLN, 15 nM) against Aurora A, PD98059 (PD, 25 nM) against Erk1/2, Wortmannin (WM, 15 nM) against phosphoinositide 3-kinase (PI3K), and BI 6727 (15 nM) against Plk1 for 24 h. The clinical information of donors is summarized in Additional file 1: Table S1, and the ASC purity was evaluated by examining the established cell surface markers for mesenchymal stem cells (MSCs) [27] depicted in Additional file 2: Table S2. As reported, primary cilia, stained by antibodies against cilium markers acetylated α-tubulin and Arl13b, in ob-ASCs were much shorter than those in ln-ASCs [11] analyzed by fluorescence microscopy (Fig. 1a, b). Further analysis showed that ln-ASCs responded only slightly to these inhibitors. While the primary cilium of untreated ln-ASCs had a mean length of 4.01 μm, it was 4.21 μm, 4.24 μm, 4.15 μm, and 3.93 μm upon the treatment with MLN, PD, WM, and BI 6727, respectively (Fig. 1a). By contrast, relative to untreated ob-ASCs (2.67 μm), the cilia of ob-ASCs extended their length after the treatment with MLN (3.90 μm, *p* < 0.01), PD (4.00 μm, p < 0.01), and WM (3.44 μm, *p* < 0.05) but not with BI 6727 (2.41 μm) (Fig. 1b). Among these four kinase inhibitors, MLN and PD worked with the best efficacy in extending the cilium length, suggesting that active Aurora A and Erk1/2 are mostly responsible for shortening cilia in ob-ASCs.

Erk is known for its roles in cell proliferation and survival in embryonic precursors [28]. All inhibitors were thus intentionally used at low concentrations, so that cell cycle progression and the sub G0 population were scarcely changed (Additional file 3: Figure S1A and B). The effectiveness of low dose (25 nM) of PD was evidenced by Western blot analyses (Additional file 3: Figure S1C). In support of the role of active Erk1/2 in ob-ASCs, the level of phosphorylated Erk1/2 (p-Erk1/2) was indeed highly increased in both types (visceral and subcutaneous) of ob-ASCs, which could be reduced to the level of ln-ASCs upon PD treatment (Additional file 3: Figure S1C). The evaluation of the fluorescence intensity of p-Aurora A (T288) proved the efficacy of 15 nM MLN treatment; the phosphorylation signal of mitotic Aurora A was significantly reduced by approximately 15% in MLN treated ob-ASCs compared to untreated ob-ASCs (Additional file 3: Figure S1D-F).

As reported [11], the cilium length of ln-ASCs was shortened to 2.90 μm by the exposure to IL6 after 24 h (Fig. 1c). In fact, both inhibitors could partially rescue this reduction (WM: 3.29 μm; PD: 3.91 μm), compared to primary cilia in control ln-ASCs with 3.99 μm (Fig. 1c, d; Additional file 4: Figure S2A). In addition, the effect of PD, the most effective inhibitor beside the Aurora A inhibitor, was further evaluated with an increased number of measured cilia from visceral and subcutaneous ob-ASCs. The addition of PD (25 nM) extended the cilium length to 4.00 μm (*p* < 0.001) in visceral ob-ASCs (ob-ASCvis) and to 4.01 μm (*p* < 0.001) in subcutaneous ob-ASCs (ob-ASCsub), relative to non-treated ob-ASCvis with 2.73 μm and ob-ASCsub with 2.91 μm (Fig. 1e–g).

As deciliation genes were highly increased in obese ASCs [11], we analyzed these genes including *AURKA* (Aurora A), *PLK1*, *PLK4*, *KIF2A*, *KIF24*, *CCP110*, and *HDAC6* in ob-ASCs after 24-h treatment with either MLN or PD inhibitor. Compared to visceral ob-ASCs, three key mitotic kinase genes, *AURKA*, *PLK1*, and *PLK4*, were significantly decreased after each inhibitor treatment (Fig. 1h, upper panel, 3 graphs of left side). HDAC6, an important tubulin deacetylase, displayed a slight increase upon PD treatment (Fig. 1h, upper panel, right graph). *CCP110*, a cilia formation regulatory gene, and two depolymerase genes *KIF2A* and *KIF24* showed no significant response to both inhibitor treatments (Fig. 1h, lower panel). In sum, these results suggest that inhibition of Aurora A and Erk1/2 with low doses of corresponding inhibitor is sufficient to rescue the length of primary cilia in ob-ASCs, together with multiple reduced deciliation genes.

### Rescued Hedgehog (Hh) signaling after low dose of MLN or PD treatment in ob-ASCs

**Fig. 2:**
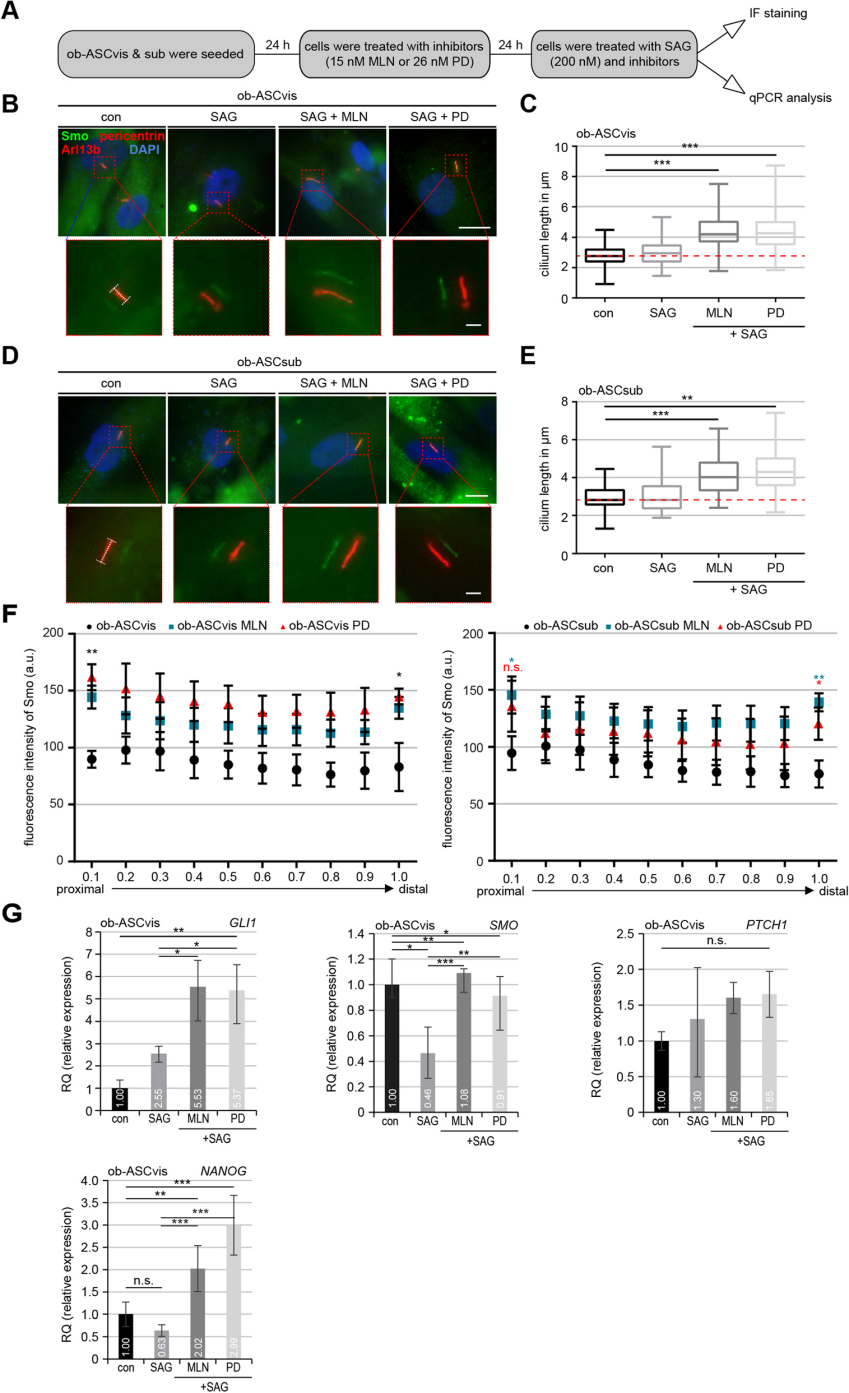
The Hedgehog pathway could be restored at protein and RNA level in primary cilia of obese ASCs by pretreatment with MLN or PD. **a** The working schedule. **b**, **d** Cells were stained against Smo (green), pericentrin (red), Arl13b (red), and DAPI (blue). Representatives are shown. Scale bar, 10 μm. Insets depict shifted overlays. Scale bar, 2.5 μm. **c**, **e** ob-ASCvis (C) and ob-ASCsub (E) were treated as indicated and stained for the evaluation of the cilium length. The results are from three experiments (*n* = 73–80 cilia for each condition in each group) and presented as median ± min/max whiskers in box plots. **f** Line-scan analyses of fluorescent Smo are shown for ob-ASCvis (left) and ob-ASCsub (right) treated with SAG and indicated inhibitors for 24 h. Each point on the graph represents the mean fluorescence intensity (mean ± SEM) based on three experiments (*n* = 30 cilia). **g** The gene levels of *GLI1*, *PTCH1*, *SMO*, and *NANOG* are shown for visceral obese ASCs treated as indicated in **a**. The results are from three experiments, presented as mean ± SEM. Unpaired Mann-Whitney *U* test for **c**, **e**, and **f**. ∗*p* < 0.05, ∗∗*p* < 0.01, ∗∗∗*p* < 0.001. Student’s *t* test for **g**

The Hh pathway is crucial for mediating intercellular communication and the development of nearly every organ in mammals [28]. It is also of particular importance for multiple differentiation processes of stem cells such as osteogenic and adipogenic differentiation [9, 29]. The activation of the Hh signaling, for instance by treatment with Smoothened agonist (SAG), recruits the pathway components Smoothened (Smo) and glioma-associated oncogene homolog 1–3 (Gli1–3) to the cilium, where these proteins accumulate on the proximal and distal tip of the cilium [28]. To investigate if these inhibitors are able to improve the Hh pathway, ob-ASCs were pretreated with MLN or PD followed by further treatment with SAG (200 nM) (Fig. 2a). Treated ob-ASCs were stained for Arl13b, Smo, and pericentrin for microscopic evaluation. The SAG stimulation has no significant effect on the cilium length (Fig. 2b–e). Compared to non-treated ob-ASCvis, the addition of MLN or PD significantly increased their cilium length to 4.34 μm and 4.36 μm, respectively (Fig. 2b, c). The comparable results were also obtained in subcutaneous ob-ASCs (Fig. 2d, e).

To investigate the Hh signaling in these cells in depth, we performed a line-scan analysis in visceral (Fig. 2f, left) and subcutaneous (Fig. 2f, right) ob-ASCs. Without the stimulation of SAG, Smo was not or diffuse localized to the cilium of ob-ASCs (Fig. 2b, d). Interestingly, upon SAG stimulation, the Smo intensity in visceral ob-ASCs was significantly increased in the proximal base (prox) and distal tip (dis) of primary cilia of MLN (prox: *p* = 0.006; dis: *p* = 0.021) as well as PD (prox: *p* = 0.005; dis: *p* = 0.017) treated cells (Fig. 2f, left). Though the response of subcutaneous ob-ASCs was not so intense as visceral ob-ASCs [11], the Smo signaling was significantly recovered in ob-ASCsub treated with MLN (prox: *p* = 0.016; dis: *p* = 0.002) or PD (prox: *p* = 0.136; dis: *p* = 0.039) (Fig. 2f, right). These results indicate that the signal transduction of the Hh pathway, evidenced by the recruitment of Smo to cilia, is significantly improved by the treatment with inhibitors, presumably by extending a minimal cilium length required for its signaling.

Moreover, the RNA was isolated from these treated cells for gene analysis. The Hh-related genes *SMO* and *GLI1* were highly increased in MLN- or PD-treated visceral ob-ASCs (Fig. 2g, upper panel). While its direct downstream target *NANOG* was significantly enhanced (Fig. 2g, lower panel, right), protein patched homolog 1 (*PTCH1*) showed no significant change upon inhibitor treatment in ob-ASCvis (Fig. 2g, lower panel, left). The similar results were also observed in subcutaneous ob-ASCs (Additional file 4: Figure S2B).

These findings indicate that MLN and PD are not only capable of restoring the cilium length of ob-ASCs, but also of regaining its function in the signal transduction like the Hh pathway.

### Ob-ASCs show increased motile capacity after restoring their cilia

**Fig. 3:**
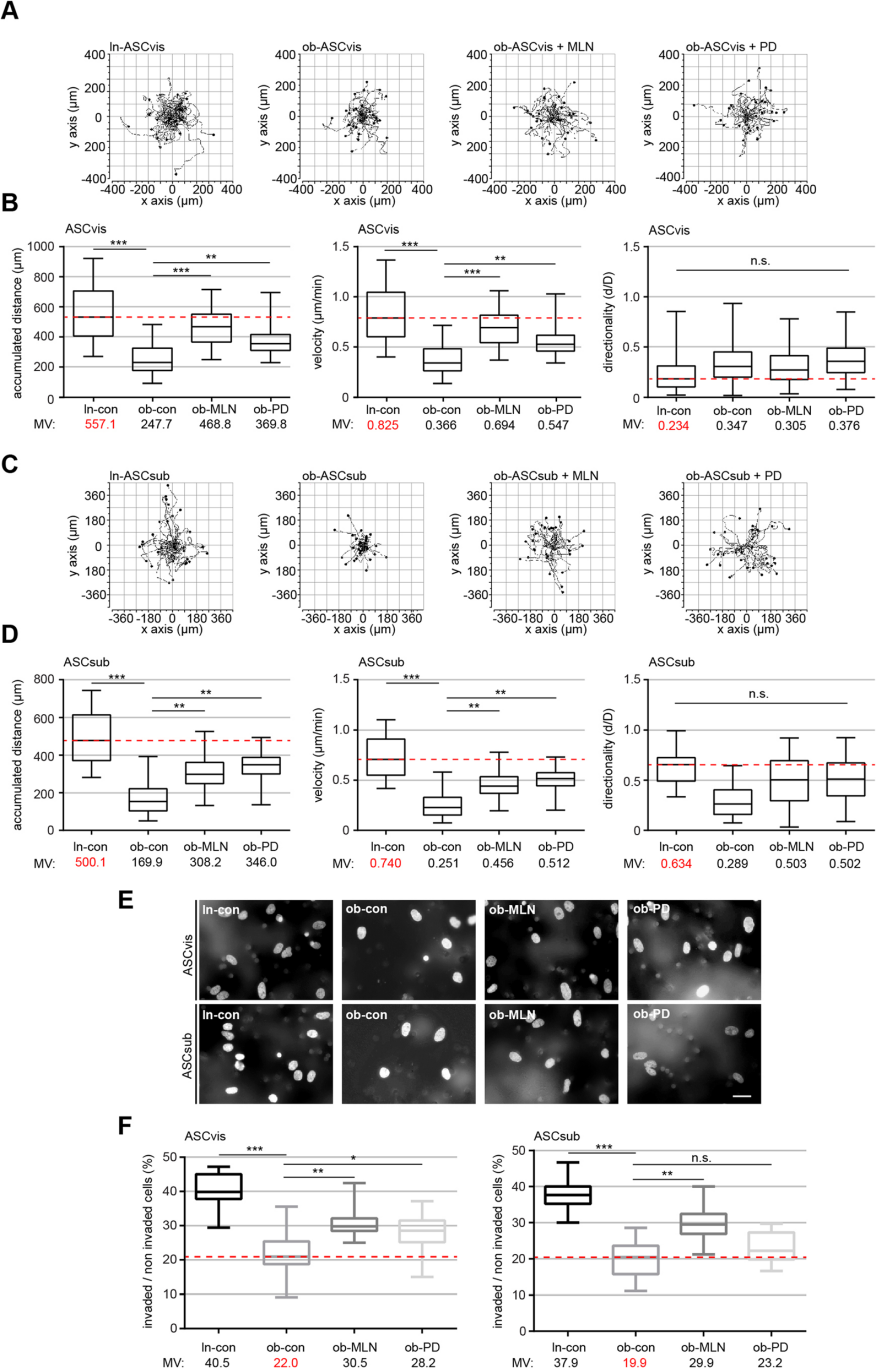
The rescue of primary cilia restores significantly the motility and invasion capacity of visceral and subcutaneous obese ASCs. **a**–**d** Analysis of cell motility of lean, obese, and obese ASCs treated with MLN or PD. Representative trajectories are depicted for individual cells (**a** and **c**, *n* = 30 cells in each group). The accumulated distance (left), the velocity (middle), and the directionality (right) are evaluated for indicated ASC as shown in the box plots (*n* = 90 cells pooled from three experiments). **e** Representatives of invaded visceral and subcutaneous ASCs (ln-con, ob-con, ob-MLN, and ob-PD) stained with DAPI. Scale bar, 30 μm. **f** Quantification of invaded ASCs. Percentage of invaded cells in comparison with the whole cell count. The results are presented as median ± min/max whiskers in box plots (visceral, left; subcutaneous, right) based on three independent experiments. An unpaired Mann-Whitney *U* test was used for statistical evaluation. ∗*p* < 0.05, ∗∗*p* < 0.01, ∗∗∗*p* < 0.001

The primary cilium is involved in cell motility, migration, and invasion [13, 30]. To examine this issue, the motility of ln-ASCs and ob-ASCs untreated or treated with MLN or PD was monitored in a single-cell tracking manner up to 12 h as reported [11, 20]. As expected, the accumulated distance was highly decreased in ob-ASCs of both subtypes (vis, 247.7 μm; sub, 169.9 μm) compared to ln-ASCs (vis, 557.1 μm; sub, 500.1 μm) (Fig. 3a–d). As calculated by time and distance, the velocity was also significantly decreased in ob-ASCs relative to their lean counterpart (Fig. 3a–d). The treatment with low dose of MLN or PD was able to rescue partially the decreased accumulated distance to 468.8 μm and 369.8 μm, respectively, in visceral ASCs, and 308.2 μm and 346.0 μm, respectively, in subcutaneous ASCs (Fig. 3a–d). Also, the velocity was significantly re-established after 24-h treatment with either of both inhibitors (Fig. 3a–d). Consistent with previous data [11, 20], visceral ASCs showed no intrinsic directionality (Fig. 3b, right graph). Their subcutaneous counterpart demonstrated a directed motility, which could be in part restored with the treatment of the inhibitors, yet not to a significant extent (Fig. 3d, right graph).

To examine the invasive potential of ln-ASCs and ob-ASCs treated or non-treated with inhibitors (MLN or PD), commonly used invasion assays were carried out [19]. 40.5% of ln-ASCvis and 37.9% in ln-ASCsub were able to invade through the matrigel layer (Fig. 3e, f). By contrast, only 22.0% of ob-ASCvis and 19.9% ob-ASCsub went through the layer (Fig. 3e, f). Intriguingly, ob-ASCs treated with MLN increased their invasion capacity up to 30.5% (ob-ASCvis) and 29.9% (ob-ASCsub), whereas 28.2% of ob-ASCvis and 23.2% of ob-ASCsub incubated with PD were capable of invading (Fig. 3e, f).

**Fig. 4:**
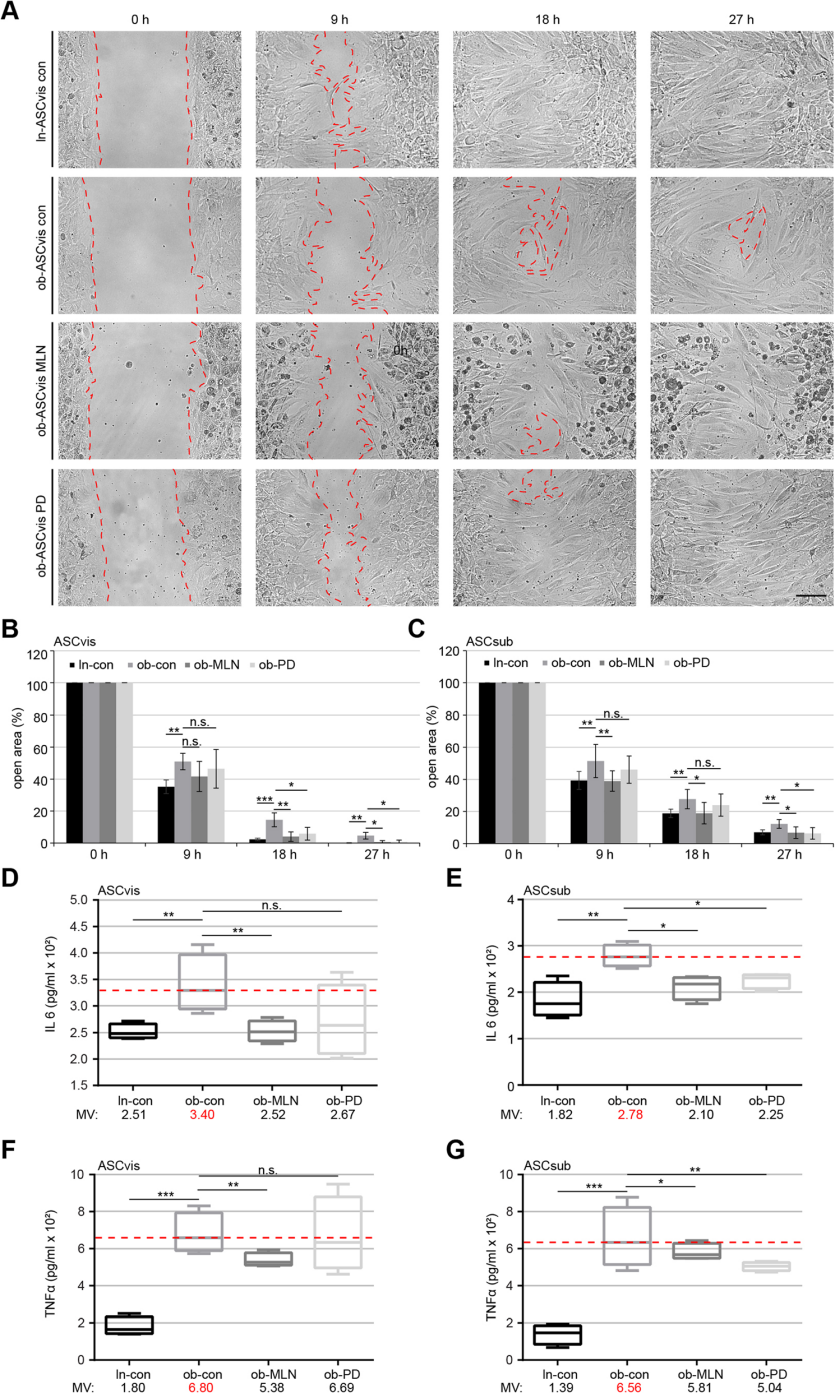
Reduced inflammatory cytokine secretion (IL6 and TNFα) and improved migration of obese ASCs after inhibitor treatment. **a** Wound healing/migration assays were performed with visceral and subcutaneous ASCs (ln-con, ob-con, ob-MLN, and ob-PD), and images were taken at indicated time points to document the migration front closure. Representatives are shown. Red dashed line depicts the migration front. Scale, 150 μm. **b**, **c** Quantification of the open area between both migration fronts at various time points is indicated (*n* = 5 visual fields of 1350 × 1800 μm^2^ for each condition). The cell-free area of each individual condition at 0 h was assigned as 100%. The results are based on three independent experiments with ASCs from three different donors (obese and lean) and presented as mean ± SEM. **d**–**g** 72-h supernatants of visceral (**d**, **f**) and subcutaneous (**e**, **g**) ASCs after 24-h pretreatment with indicated inhibitors were collected for the evaluation of IL6 (**d**, **e**) and TNFα (**f**, **g**). The results are from three experiments and presented as median ± min/max whiskers in box plots. Student’s *t* test was used for statistical evaluation for (**b**–**g**). ∗*p* < 0.05, ∗∗*p* < 0.01, ∗∗∗*p* < 0.001

To study if cell migration is restored after the treatment with PD or MLN, wound healing/migration assays were performed [19]. After 18 h and 27 h, ob-ASCs treated with MLN or PD showed a significantly increased migration capacity compared to non-treated visceral (Fig. 4a, b) and subcutaneous ob-ASCs (Fig. 4c). However, the effect of PD was weaker than MLN, despite both inhibitors had comparable effects on restoring the cilium length. This could be ascribed to the importance of the mitogen-activated protein kinase (MAPK/Erk) signaling in migration [31]. Nevertheless, both inhibitors could improve the motility of ob-ASCs, probably by restoring the primary cilium function with its multiple connections in migration and invasion signaling pathways.

### MLN or PD alters the secretion of inflammatory cytokines of ob-ASCs

The primary cilium is connected to the secretion of inflammatory cytokines [11, 32]. To investigate if the inhibitor treatment affects the secretion, we measured IL6 and TNFα concentrations in the supernatant of ASCs. Compared to untreated ob-ASCs, MLN-treated ob-ASCs significantly reduced the secretion of both cytokines (Fig. 4d–g). Though a clear reduction, the effect of PD on the secretion of visceral ob-ASCs was not significant due to a high standard derivation (Fig. 4f). These results strengthen the notion that these kinase inhibitors are able to decrease the secretion of pro-inflammatory cytokines by rescuing their primary cilia and possibly also by impacting the gene and protein levels of these cytokines.

### MLN or PD enhances osteogenic and adipogenic differentiation of ob-ASCs

**Fig. 5:**
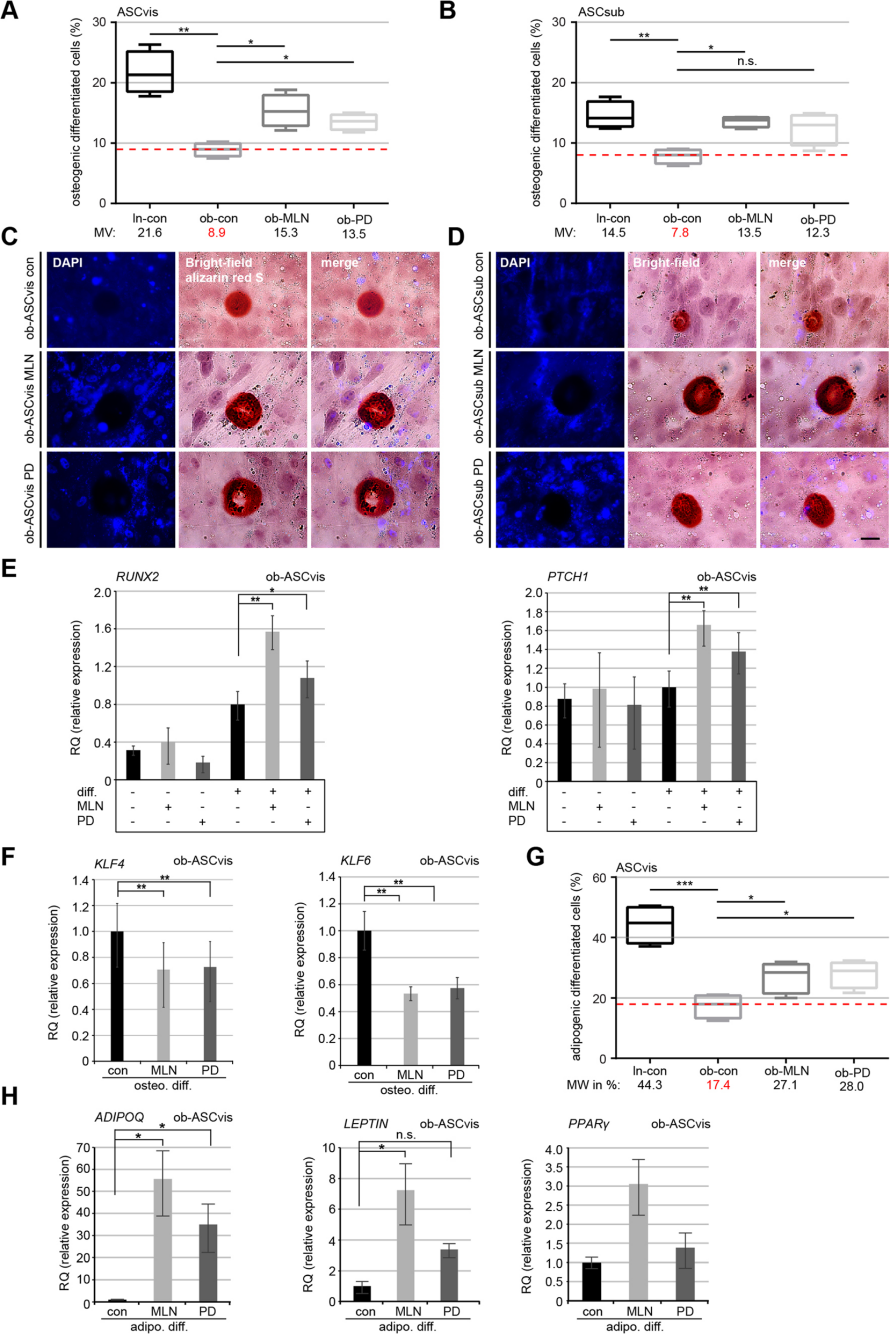
Enhanced osteogenic and adipogenic differentiation ability in obese ASCs treated with MLN or PD accompanied by downregulation of the transcription factors *KLF4* and *KLF6.* **a**–**d** Obese ASCs, pretreated for 72 h with MLN or PD, were induced into the osteogenic differentiation. The percentage of differentiated ASCs was evaluated by alizarin red S staining. **a**, **b** The results are presented as median ± min/max whiskers (red dashed line indicates median value of obese ASCs) in visceral and subcutaneous ASCs (*n* = 300 cells for each condition, pooled from three experiments). Example images are shown in **c** and **d**, respectively. Scale bars 30 μm. **e** Gene analyses of visceral ASCs with indicated conditions for *RUNX2* and *PTCH1*, two genes involved in osteogenesis. The results are from three experiments and presented as mean ± SEM. **f** Gene levels of *KLF4* and *KLF6* after osteogenic differentiation in visceral obese ASCs. The results are from three experiments, presented as mean ± SEM. **h** Gene Levels of *ADIPOQ*, *LEPTIN*, and *PPARγ* after adipogenic differentiation are shown for obese visceral ASCs. The results are from three experiments, presented as mean ± SEM. **g** Quantification of cells showing lipid vacuoles after 14 days of adipogenic differentiation. The results are presented as median ± min/max whiskers in visceral ASCs (*n* = 150 cells for each condition, pooled from three experiments), and the red dashed line illustrates the median value of obese ASCs. Box and whisker plots were used to show the median and the minimal to maximal range of the values in **a**, **b**, and **g**. Unpaired Mann-Whitney *U* test for **a**, **b**, and **g**. Student’s *t* test for **e**, **f**, and **h**. ∗*p* < 0.05, ∗∗*p* < 0.01, ∗∗∗*p* < 0.001

We reported that ob-ASCs highly reduced their known adipogenic and osteogenic differentiation capacity [33], which could also be induced in ln-ASCs by shortening primary cilia with IL6 treatment [11]. To investigate if extended primary cilia could improve the differentiation ability of ob-ASCs, pretreated or non-pretreated visceral ob-ASCs were induced to differentiate to osteocytes (Fig. 5a–d) or adipocytes (Fig. 5g, h). The quantification of alizarin red S, used to visualize calcium deposition, revealed that 72 h pretreatment with MLN or PD increased significantly the osteogenic differentiation capacity of visceral ob-ASCs to 15.3% and 13.5%, respectively, relative to 8.9% in non-treated ob-ASCs and 21.6% in ln-ASCs (Fig. 5a, c). Similar results were also obtained from subcutaneous ob-ASCs, except the PD treatment did not reach a significant extent (*p* > 0.0571) (Fig. 5b, d). These results suggest that only 3-day inhibitor treatment is able to partially rescue the differentiation capacity of ob-ASCs.

Moreover, we analyzed the gene levels of known osteogenic markers *RUNX2* and *PTCH1,* and transcription factors *KLF4* and *KLF6*, which are related to osteogenic differentiation [34]. Both osteogenic marker genes *RUNX2* and *PTCH1* were significantly elevated in visceral ob-ASCs treated with MLN or PD compared to untreated ob-ASCs (Fig. 5e). The gene level of *KLF4,* negatively involved in the osteogenic differentiation of mesenchymal stem cells [34], was significantly downregulated in ob-ASCs treated with MLN or PD (Fig. 5f, left graph). Additionally, *KLF6*, another member of the *KLF* transcription factor family, was also decreased in these treated ob-ASCs (Fig. 5f, right graph). Subcutaneous ob-ASCs showed comparable results as visceral ob-ASCs, except the gene level of *RUNX2*, were not increased after inhibitor treatment (Additional file 4: Figure S2C), and the overall response to the induced osteogenic differentiation was reduced compared to visceral ob-ASCs (Fig. 5a and c vs b and d). Altogether, the inhibition of either Aurora A by MLN or Erk1/2 by PD was able to increase the osteogenic differentiation potential of obese visceral and subcutaneous ASCs likely by the downregulation of several downstream transcription factors like *KLF4* and *KLF6*.

These data were further corroborated by an adipogenic differentiation assay. Microscopic examination revealed a highly increased number of differentiated cells displaying lipid vacuoles with 27.1% in MLN-treated visceral ob-ASCs and 28.0% in PD-treated ob-ASCs, compared to 17.4% in non-treated ob-ASCs (Fig. 5g). In support of these observations, the gene levels of *ADIPOQ*, *LEPTIN*, and *PPARγ* were highly elevated in 72-h pretreated ob-ASCs (Fig. 5h). Both inhibitors are thus able to enhance the osteogenic and adipogenic differentiation potential of ob-ASCs.

### MLN or PD increases the expression of multiple self-renewal-associated genes in ob-ASCs

**Fig. 6:**
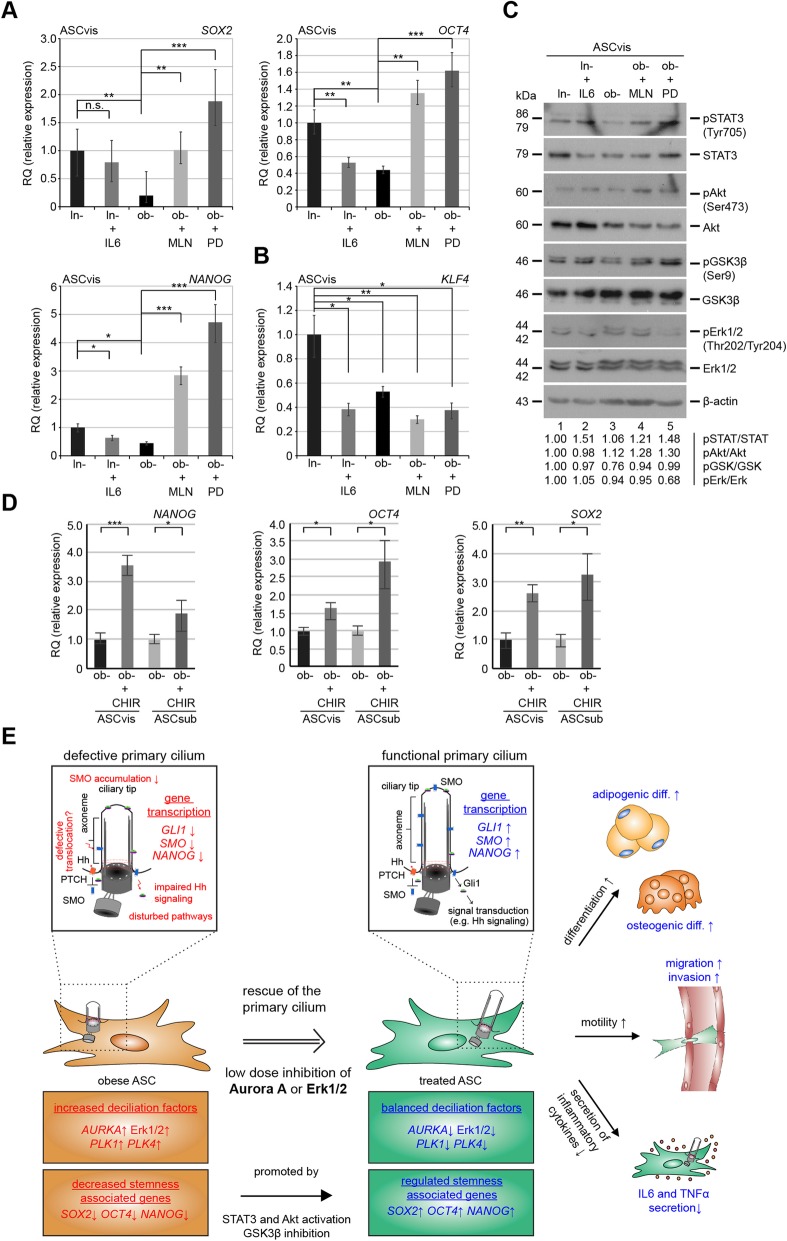
The inhibition of Aurora A and Erk1/2 increases the level of stemness-associated genes including *SOX2*, *OCT4*, and *NANOG* and modulates the STAT3-AKT/GSK3β signaling in obese ASCs. **a**, **b** The gene levels of genes associated with pluripotency (*SOX2*, *OCT4*, *NANOG*, and *KLF4*). The data are based on four experiments and presented as mean ± SEM. Student’s *t* test was used. ∗*p* < 0.05, ∗∗*p* < 0.01, ∗∗∗*p* < 0.001. **c** Western blot analyses of pSTAT3, STAT3, pAKT, AKT, pGSK3β, GSK3β, pErk1/2, Erk1/2, and β-actin. The intensity of proteins was normalized to β-actin, and ratios were quantified with image J. Lean ASCvis were set as 1. **d** The gene levels of *SOX2*, *OCT4*, and *NANOG* in obese ASCs untreated or treated with a GSK3β inhibitor (CHIR99021, 2 μM) for 48 h. The data are based on three experiments and presented as mean ± SEM. Student’s *t* test was used. ∗*p* < 0.05, ∗∗*p* < 0.01, ∗∗∗*p* < 0.001. **e** Schematic illustration of the proposed working model. Obese ASCs with shortened primary cilia, which render these cells dysfunctional, can be partly rescued by treatment with low dose of Aurora A or Erk1/2 inhibitors. This improves their stemness, motility, and differentiation capacity and modifies their cytokine secretion

The MAPK/Erk signaling as well as the Aurora A activity was shown to be associated with the differentiation of human embryonic stem cells (hESCs) and induced pluripotent stem cells (iPSCs) by interacting with the glycogen synthase kinase 3 beta (GSK3β), an antagonist of Wnt signaling [35–37]. Indeed treated ob-ASCvis showed a significantly increased expression of stemness/self-renewal-associated genes like *SOX2*, *OCT4*, and NANOG compared to non-treated ob-ASCs and ln-ASCs treated with IL6 (Fig. 6a). Moreover, the gene level of *KLF4* was decreased in MLN- and PD-treated ob-ASCs (Fig. 6b). *KLF4* is a downstream target of the MAPK/Erk signaling pathway [38] and is probably also regulated by Aurora A (Fig. 5f). Interestingly, the treatment changed hardly the expression of *c-MYC* (Additional file 4: Figure S2D).

Moreover, both inhibitors were able to activate the signal transducer and activator of transcription 3 (STAT3) and phosphatidylinositol 3-kinase (PI3K/AKT) pathway by showing increased levels of pSTAT3 (Tyr 705) and pAkt (Ser473) (Fig. 6c, 1st to 4th row, lanes 4 and 5) compared to untreated ob-ASCs (Fig. 6c, 1st to 4th row, lane 3). Importantly, MLN- and PD-treated cells showed an increase in the inhibitory phosphorylation of GSK3β (Ser 9) (Fig. 6c, 5th and 6th row, lane 3 vs 4 and 5), which is known to upregulate several transcription factors involved in self-renewal and stemness [39]. This was further corroborated by using CHIR99021, a specific inhibitor against GSK3β [40]. Both types of ASCs displayed a significant increased gene expression of *NANOG*, *OCT4*, and *SOX2* compared to untreated obese ASCs (Fig. 6d). These data strengthen the notion that improved differentiation capability of ob-ASCs by using these two inhibitors is also mediated by GSK3β, a crucial regulator in maintaining the self-renewal/stemness of mesenchymal stem cells.

## Discussion

We have recently reported that obese ASCs exhibit short and dysfunctional primary cilia [11]. In the present work, we show that multiple inhibitors targeting cilium destabilizing kinases and other pathways are able to elongate the ciliary length and partially rescue the functionalities of obese ASCs. In particular, low dose of Aurora A inhibitor MLN or Erk1/2 inhibitor PD was able to improve the Hh signaling at the primary cilium, the motility, and differentiation capacity and to reduce the secretion of inflammatory cytokines of obese ASCs. These results highlight that restoring the impaired primary cilia ameliorate the functionalities of obese ASCs, which might slow down the progression of diseased adipose tissues in obesity.

The primary cilium is tightly associated with the cell cycle [13] regulating cell proliferation during normal and cancer development [14]. Ciliogenesis is tightly coordinated by diverse cell cycle-coupled kinases like Aurora A and Plk1 initiating ciliary disassembly or blocking its extension [13], which were reported to be upregulated at the gene level of obese ASCs [11]. Furthermore, obesity is commonly characterized by an increased pro-inflammatory milieu [3, 41]. IL6 is known to activate a variety of pathways such as STAT3, MAPK, and PI3K signaling as well [42]. It is therefore conceivable that inhibition of kinases like Aurora A and Erk1/2 are able to prolong the length and functionalities of primary cilia in ASCs derived from obese patients. Indeed, we show here that Aurora A and Erk1/2 are the most effective targets for restoring ciliary length. Low dose of both inhibitors against deciliation kinases is sufficient to stabilize the primary cilium in obese ASCs without a significant interference with cell cycle progression. Interestingly, inhibition of these kinases reduces gene levels of important ciliary disassembly factors like *AURKA*, *PLK1*, and *PLK4*, suggesting the involvement of these kinases in regulating the gene expression, possibly indirectly.

Interestingly, a great body of recent studies has focused on the relationship between ciliary signaling pathways and cancer development [43, 44]. Moreover, multiple kinase inhibitors, which are in preclinical or clinical trials for tumor therapy, could have off-target effects by interfering with the ciliation in non-tumor cells mediated through the regulation of Aurora A activity [45]. In line with this notion, we report here that the Aurora A inhibitor MLN affects indeed the ciliation of ASCs. In addition, Plk1 is highly linked to cancer development: activated Plk1 induces a rapid loss of ciliation by binding to Dishevelled 2 (Dvl2) or by phosphorylation of nephrocystin-1 in HeLa and hTERT-RPE cell lines [46, 47], raising the question whether Plk1 inhibitors like volasertib (BI 6727) used in clinical trials are able to interfere with ciliogenesis [48]. In this context, our data indicate that low dose of Plk1 inhibitor BI 6727 is not able to modulate ciliary length, at least in ASCs.

The inhibition of deciliation kinases enhances greatly the differentiation potential of obese ASCs, which could be explained by following alterations. First, inhibition of Aurora A or Erk1/2 improves the Hh signaling at the primary cilium in ob-ASCs. The evolutionarily conserved Hh pathway is involved in embryonic development, stem cell maintenance [15, 49, 50], and osteogenic differentiation of ASCs [51]. The restored Hh pathway contributes thus directly to the increased adipogenic and osteogenic differentiation capacity in MLN- or PD-treated obese ASCs. Second, the inhibitors decrease the expression of *KLF4*, a negative regulator for differentiation in MSCs [52], facilitating the differentiation of treated obese ASCs. Third, the treatment with MLN or PD significantly increased stem cell/self-renewal-related genes like *OCT4*, *NANOG*, and *SOX2*, providing a further molecular explanation for the improved differentiation capacity of obese ASCs.

Our observation showing increased stem cell/self-renewal genes after the treatment with MLN is supported by a study where the inhibition of Aurora A enhances the reprogramming efficiency of induced pluripotent stem cells (iPSCs) linked to increased gene levels of *NANOG*, *TET1*, and *ERas*, mediated by GSK3β inactivation [35], which is necessary to promote iPSC generation [53]. Moreover, human pluripotent stem cells (hPSCs) require the PI3K/AKT activity to maintain the self-renewal state by suppression of the MAPK/Erk and canonical Wnt pathways [36, 37, 54]. The induction of active pErk1/2 was further connected to reduced *NANOG* gene levels [37]. In line with these data from iPSCs and hPSCs [36, 37, 54], we show an enhanced expression of stemness/self-renewal-related genes in obese ASCs after inhibition of either Aurora A or Erk1/2. Both inhibitors lead to the activation of the STAT3 and PI3K/AKT pathways, which phosphorylates its downstream target GSK3β on serine 9 [55]. This phosphorylation is within the substrate binding site of GSK3β and thus hinders the interaction with its binding partners and prevents the phosphorylation of multiple downstream targets [55, 56]. As reported in embryonic stem cells (ESCs) [57], the inactivation of GSK3β resulted in increased *NANOG* transcription and stemness. Intriguingly, IL6 stimulated through the phosphorylation of STAT3 but showed no impact on Akt or GSK3β, as reported in HepG2 cells [58]. Altogether, inhibition of Aurora A or Erk1/2 enhances the differentiation ability of obese ASCs by improving the Hh signaling pathway, decreasing the expression of *KLF4* and increasing the gene level of self-renewal/stemness-associated genes. Additionally, the direct inhibition of GSK3β in obese ASCs emphasizes the role of the canonical WNT signaling in maintaining stemness even in adult mesenchymal stem cells.

## Conclusion

In summary, this work demonstrates that the treatment with low dose of the Aurora A inhibitor MLN8054 or Erk1/2 inhibitor PD98059 is able to partially restore the functionalities of obese ASCs by stabilizing their primary cilia and reestablishing a balance of multiple stemness/self-renewal- and ciliary-associated genes (Fig. 6e). These restored ASCs might improve adipogenesis, hypoxia, and impaired immunomodulation in obese tissues [59, 60] and consequently slow down morbid obesity-associated diseases. Furthermore, autologous MSCs including ASCs provide a novel therapeutic strategy in a wide spectrum of diseases [61], and enabling the use of patient’s own ASCs will support engraftment rates by reducing the chance of immune rejection [62]. Further studies in vitro and in vivo are required to gain further insights into the reciprocal influence of obesity and dysfunctional ciliogenesis.

## Additional files

Additional file 1: **Table S1.** Clinical information of 18 patients. (DOCX 22 kb)

Additional file 2: **Table S2.** Cell surface markers of ASCs. (DOCX 19 kb)

Additional file 3: **Figure S1.** Comparable cell cycle distribution between control and MLN- or PD-treated ASCs, and proof of concept for low-dose treatments. (JPG 855 kb)

Additional file 4: **Figure S2.** Inhibition of Erk1/2 or Aurora A rescues the Hh signaling pathway and the osteogenic differentiation capacity of subcutaneous ASCs. (JPG 549 kb)

## Data Availability

Not applicable.
