# Supplementary material for: Restoration of primary cilia in obese adipose-derived mesenchymal stem cells by inhibiting Aurora A or extracellular signal-regulated kinase
Source: Stem Cell Res Ther. 2019 Aug 14;10:255. doi: 10.1186/s13287-019-1373-z (PMC6694567; doi:10.1186/s13287-019-1373-z)
Supplement: Supplementary file 1 — Table S1. Clinical information of 18 patients. (DOCX 22 kb) [file 13287_2019_1373_MOESM1_ESM.docx]

| patients | age | gestational age (weeks) | body mass index  (BMI) | birth weight (g) |
| --- | --- | --- | --- | --- |
| lean (n=8) | 31.0 ± 5.9 | 39.2 ± 2.6 | 22.6 ± 2.8 | 3237 ± 702 |
| obese (n=10) | 31.7 ± 6.4 | 36.0 ± 3.7 | 37.9 ± 4.5 | 3115 ± 971 |

**Table S1. Clinical information of 18 patients**
