# Supplementary material for: Restoration of primary cilia in obese adipose-derived mesenchymal stem cells by inhibiting Aurora A or extracellular signal-regulated kinase
Source: Stem Cell Res Ther. 2019 Aug 14;10:255. doi: 10.1186/s13287-019-1373-z (PMC6694567; doi:10.1186/s13287-019-1373-z)
Supplement: Supplementary file 2 — Table S2. Cell surface markers of ASCs. (DOCX 19 kb) [file 13287_2019_1373_MOESM2_ESM.docx]

**Table S2. Cell surface markers of ASCs**

|  | Cell surface markers (in %) | | | | | | | |
| --- | --- | --- | --- | --- | --- | --- | --- | --- |
|  | **CD90** | **CD73** | **CD105** | **CD146** | **CD14** | **CD31** | **CD34** | **CD106** |
| ln-ASCvis | 85.75 | 87.33 | 95.30 | 84.95 | 4.41 | 1.72 | 1.57 | 7.57 |
| ± SEM | 10.54 | 14.27 | 17.90 | 1.49 | 5.04 | 0.89 | 1.86 | 3.13 |
| ln-ASCsub | 86.48 | 89.57 | 76.08 | 96.16 | 4.55 | 4.42 | 0.85 | 5.81 |
| ± SEM | 8.44 | 7.86 | 12.34 | 0.49 | 6.01 | 3.15 | 1.06 | 0.22 |
| ob-ASCvis | 91,83 | 90,35 | 88,21 | 94,12 | 2,14 | 3,76 | 2,14 | 34,52 |
| ± SEM | 4,72 | 10,55 | 15,20 | 6,16 | 2,61 | 2,46 | 2,04 | 16,75 |
| ob-ASCsub | 91,33 | 88,84 | 82,82 | 94,32 | 0,95 | 3,23 | 1,82 | 2,42 |
| ± SEM | 2,87 | 4,36 | 16,15 | 4,22 | 0,41 | 1,95 | 1,47 | 1,73 |

Mean values representing the percentage of ASCs expressing different surface specific markers (n = 7 different patient samples for each subgroup). ASCvis, visceral adipose-derived stem cells; ASCsub, subcutaneous adipose-derived stem cells; SEM, standard error of the mean; ln, lean; ob, obese.
