# Supplementary figures and images for: Restoration of primary cilia in obese adipose-derived mesenchymal stem cells by inhibiting Aurora A or extracellular signal-regulated kinase
Source: Stem Cell Res Ther. 2019 Aug 14;10:255. doi: 10.1186/s13287-019-1373-z (PMC6694567; doi:10.1186/s13287-019-1373-z)

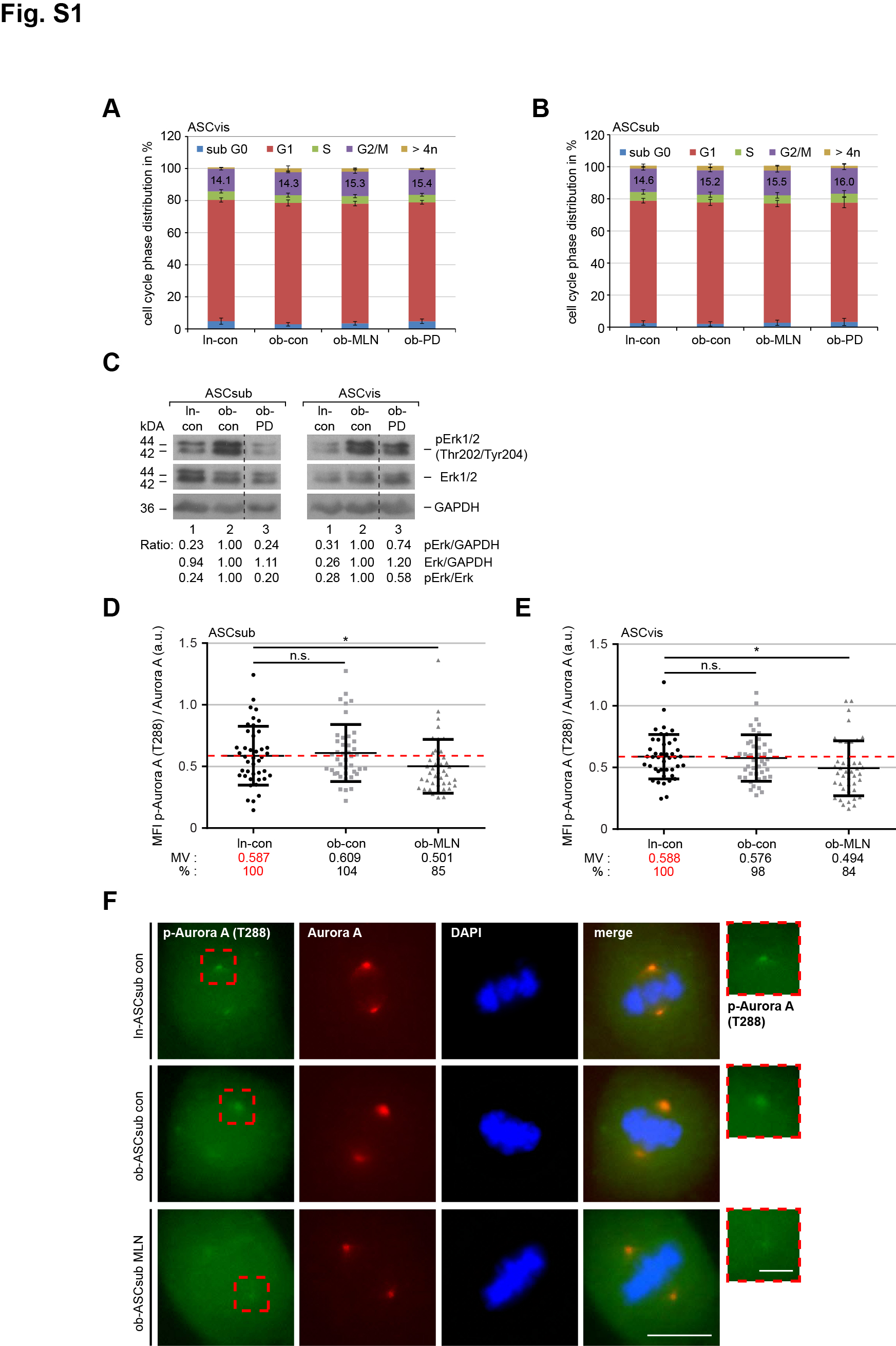

Supplement: Supplementary file 3 — Figure S1. Comparable cell cycle distribution between control and MLN- or PD-treated ASCs, and proof of concept for low-dose treatments. (JPG 855 kb) [file 13287_2019_1373_MOESM3_ESM.jpg]

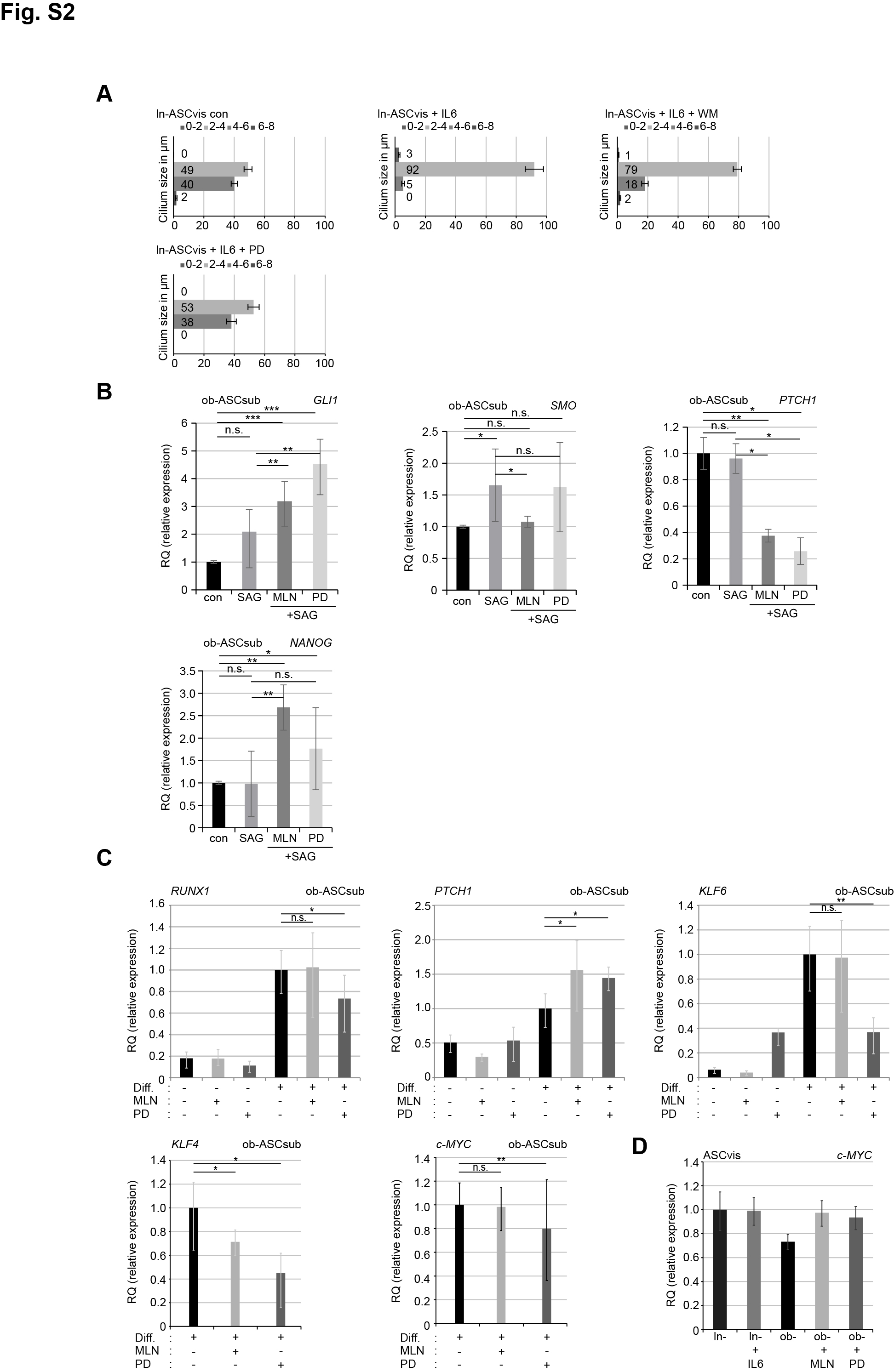

Supplement: Supplementary file 4 — Figure S2. Inhibition of Erk1/2 or Aurora A rescues the Hh signaling pathway and the osteogenic differentiation capacity of subcutaneous ASCs. (JPG 549 kb) [file 13287_2019_1373_MOESM4_ESM.jpg]
